# Differences in Bone Mineral Density and Hip Geometry in Trochanteric and Cervical Hip Fractures in Elderly Chinese Patients

**DOI:** 10.1111/os.12456

**Published:** 2019-04-26

**Authors:** Ming Li, Hou‐chen Lv, Jian‐heng Liu, Xiang Cui, Guo‐fei Sun, Jian‐wei Hu, Li‐cheng Zhang, Pei‐fu Tang

**Affiliations:** ^1^ Department of Orthopaedics General Hospital of Chinese PLA Beijing China

**Keywords:** Bone mineral density, Cervical hip fractures, Hip geometry, Trochanteric fractures

## Abstract

**Objective:**

To assess the differences in bone mineral density (BMD) and hip geometry in trochanteric and cervical hip fractures in elderly Chinese patients.

**Methods:**

A consecutive series of 196 hip fracture patients aged over 50 years was recruited from November 2013 to October 2015, including 109 cases of cervical fractures (36 males and 73 females) and 87 cases of trochanteric fractures (34 males and 53 females). All patients were evaluated through dual‐energy X‐ray absorptiometry, and baseline characteristics, BMD and structural parameters were collected and reviewed.

**Results:**

There were statistically significant differences in age, height, and body mass index between patients with each type of fracture, and patients with trochanteric fractures were older than those with cervical fractures, especially in women. The BMD in trochanteric fractures was markedly lower than in cervical fractures in all five sites of the hip by an approximate reduction of 10%, in both men and women. The cross‐sectional area, cross‐sectional moment of inertia, and the cortical thickness in the cervical fracture group were significantly higher than in the trochanteric fracture group. However, the buckling ratio of both the femoral neck and trochanteric region were significantly lower in the cervical fracture group. Age (/10 years), cross‐sectional moment of inertia in femoral neck and buckling ratio in trochanteric region were significant risk factors for trochanteric fractures compared with cervical fractures.

**Conclusions:**

Compared with cervical hip fractures, patients with trochanteric fractures were older, had a lower BMD, and had less bone mechanical strength, especially in female patients. Age, femoral neck cross‐sectional moment of inertia (FNCSMI), and trochanteric region buckling ratio (ITBR) were stronger risk factors for trochanteric hip fractures than for cervical fractures.

## Introduction

As the most serious complication of osteoporosis, the incidence of hip fractures has increased rapidly in recent decades^1^. With the aging process, the number of hip fractures worldwide may soar to 6.26 million by 2050^2^, of which over 50% will be in Asia^3^. Hip fracture is a potentially deadly disease, with high morbidity and mortality rates^4^, and a hip fracture is associated with a 2.5‐fold increased risk of subsequent fracture, which is not entirely explained by prefracture risk factors^5^. 27.6%‐40.5% of men and 15.8%‐23.3% of women will die within the first year, and nearly 40% of patients may lose their mobility and need long‐term care^6^, which results in a high economic burden for both patients and society, as well as significant disability for individuals^7^, ^8^.

The most well‐known risk factors for hip fractures are low bone mineral density (BMD) and variation in hip geometry. The risk of hip fracture increases 2.6‐fold with every decrease of one standard deviation in the BMD of the femoral neck^9^. Changes in hip geometry, including longer hip axis length (HAL), larger neck shaft angle (NSA), and a greater femoral neck width, may increase the risk of hip fracture^10^. Each centimeter increase in HAL increases the risk of hip fracture by 50% to 80% in elderly white women, and an increase of one standard deviation in NSA is associated with an odds ratio (OR) of hip fracture of 2.45 in men and 3.48 in women^11^. However, because of differences in anatomical morphology, bone content, and cortical and cancellous bone distribution, the risk factors vary for the two types of hip fracture referred to as cervical (intracapsular) fractures and trochanteric (extracapsular) fractures. Some researchers have suggested that BMD and hip geometry differ in these two types of fracture^12^. Patients with trochanteric fractures had a lower BMD and shorter HAL compared with those with femoral neck fractures^13^. The OR for fractures decreased when the cross‐sectional area (CSA) and neck length of the femur increased 1.97 times and 1.73 times in femoral neck fractures, respectively, and the OR for fractures increased when the femoral neck width increased 1.53‐fold. In addition, the OR for fractures increased when the femoral neck width increased 1.45‐fold in trochanteric fractures^14^. However, Li *et al* 15. found that there were no significant differences in BMD and hip geometry in femoral neck fractures and trochanteric fractures. Therefore, the role of BMD and hip geometry remains controversial. In addition, BMD and hip geometry vary depending on race and gender. Compared with other races, Asians have thicker cortical bone and lower buckling ratios, which may partially explain the lower prevalence of hip fractures^16^, ^17^.

Nevertheless, studies of BMD and hip geometry in Asians are rare and little is known about differences in BMD and hip geometry in different types of hip fracture. To further distinguish between the two fracture types in Asians, our study investigates the variation between trochanteric and cervical hip fractures in elderly Chinese patients in terms of BMD and hip geometry. The results may help in devising follow‐up treatments and aiding in prevention of the two types of hip fracture.

## Materials and Methods

### *Subjects*

**Figure 1:**
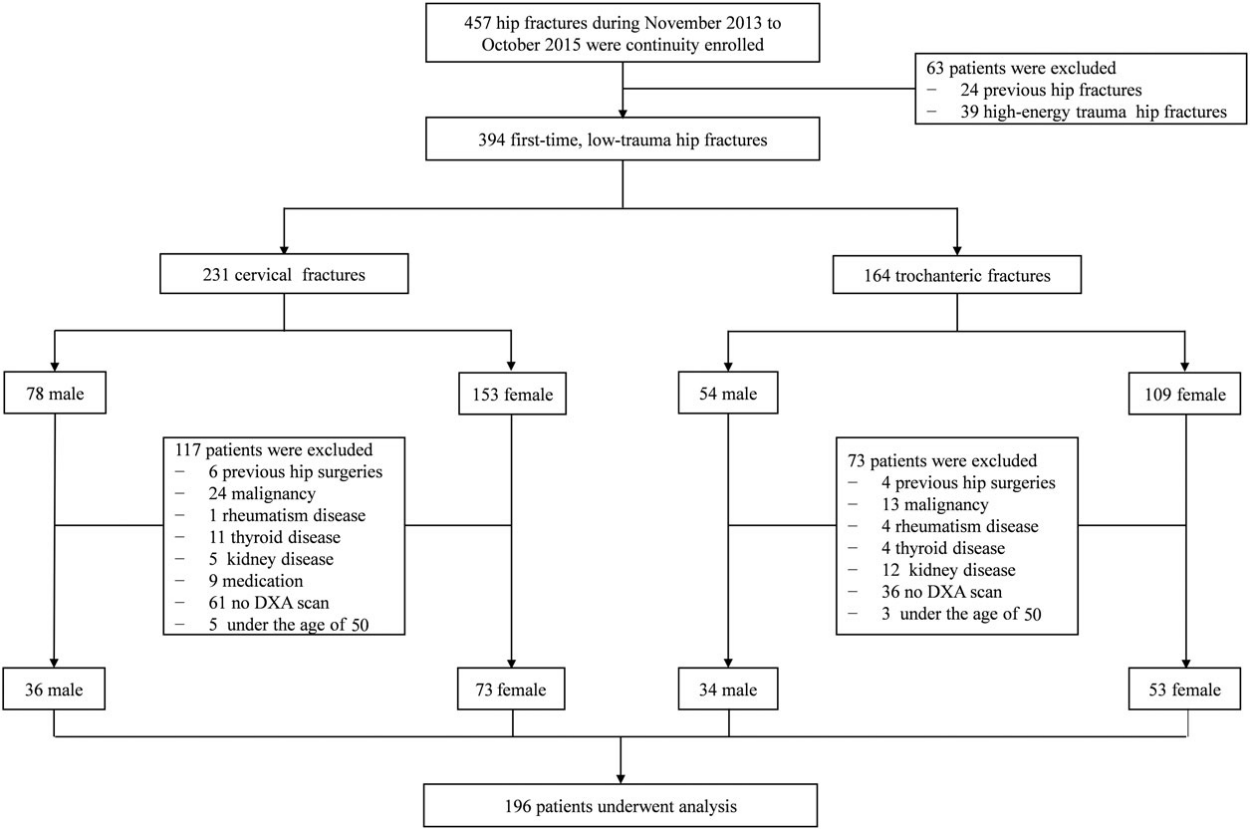
Flow chart describing the process of patient enrollment.

A total of 457 patients with a hospital diagnosis of femoral neck fracture or femoral intertrochanteric fracture (International Classification of Diseases, 9th edition, code: 820) were admitted to the Chinese PLA general hospital between November 2013 and October 2015. All patients were screened using strict inclusion and exclusion criteria. The inclusion criteria is patients with first‐time, low‐trauma hip fractures; a low‐trauma hip fracture is defined as a fracture of the proximal femur caused by an injury equal to or less than a fall at standing height. The exclusion criteria include: (i) patients were under the age of 50; (ii) patients had not been examined using dual energy X‐ray absorptiometry (DXA); (iii) patients had undergone previous surgeries on fractured hips; and (iv) patients had malignancy, rheumatoid arthritis, or metabolic bone disease. Based on these criteria, 49.7% of patients (n = 196) were finally included in the study after screening (Fig. 1). All patients were evaluated through DXA. Baseline characteristics, such as age, sex, menopause age, height, weight, and body mass index (BMI) were collected at admission.

### *Bone Mineral Density Measurement*

Bone mineral density measurements (g/cm^2^) were performed at the hip through DXA (HOLOGIC Discovery‐A, Apex software version 13.3, Bedford, MA, USA) by trained personnel using equal measurement routines. The standard position was used and the scanned image met the following criteria^18^: (i) hip joint in the center of the image; (ii) femur shaft perpendicular to the transverse plane with 15°–25° internal rotation; (iii) femur neck, head, and greater trochanter are shown completely in the image; and (iv) some soft tissue is present in the lateral femur shaft. The measurement of the patients was performed 1–2 days after admittance to hospital before the treatment. Five parts of the hip (at the non‐fracture side of the fracture patients) were measured at the following sites: femoral neck, trochanter, inner, Ward’s triangle, and total hip. The investigated parameters included the BMD (g/cm^2^), bone mineral content (g), projected area (cm^2^), and T‐score, which were all generated automatically. The coefficient of variation for the total hip BMD was 1%.

### *Hip Geometry Measurement*

**Figure 2:**
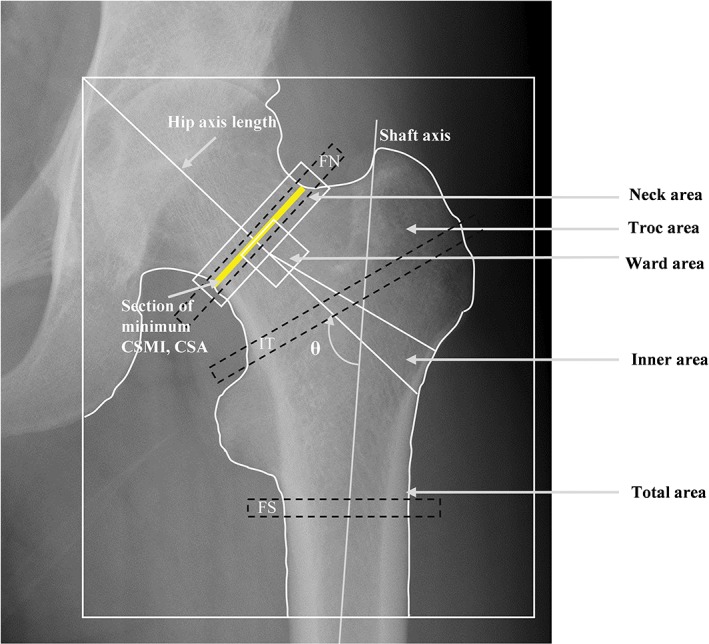
Bone mineral density was measured at five parts of the hip (non‐fracture side of the fracture patients), including the femoral neck, trochanter, inner, ward’s triangle, and total hip. Hip structural analysis was performed across the cross‐section of three different sites (dotted black frame) including the femoral neck (FN), trochanteric region (IT), and femoral shaft (FS).

Using software provided by the DXA manufacturer, hip geometry and hip structural analysis (HSA) were assessed across the cross‐section of three different sites as follows: femoral neck (FN), trochanteric region (IT), and femoral shaft (FS). Parameters measured at these three sites included subperiosteal width (SubPeriWidth), estimated endosteal width (EndoCortWidth), cross‐sectional area (CSA), cross‐sectional moment of inertia (CSMI), section modulus (Z), estimated cortical thickness (CortThick), buckling ratio (BR), and neck shaft angle and hip axis length (NSA and HAL) (Fig. 2)^19^, ^20^.

### *Statistical Analyses*

Predictor variables were chosen based on their association with hip fracture from previous studies and the above results. All data were expressed as mean and standard deviation of the mean using SPSS 22.0 software (IBM Corporation, Armonk, NY, US). The univariate significance of the comparison between the two types of hip fracture was established through χ^2^‐tests for categorical variables and through a *t*‐test for continuous variables. To detect potential associations between predictor variables and types of hip fracture, OR and 95% confidence intervals (CI) computed through logistic regression were used as measures of association. *P* < 0.05 was defined as significant in all tests.

## Results

### *Baseline Characteristics*

On the basis of the inclusion and exclusion criteria, a total 196 patients with hip fractures were included in our research for further analysis. There were 109 cervical fractures (36 men and 73 women) and 87 trochanteric fractures (34 men and 53 women). Baseline characteristics, including age, sex, menopause age, height, weight, and BMI are presented in Table 1. The average age of the patients was different in the two groups (cervical and trochanteric), at 75.00 ± 9.47 years and 78.72 ± 8.49 years (*P* = 0.002), respectively. Patients with trochanteric fractures were older than those with cervical fractures, especially in women. The height and BMI of female patients with cervical fractures were lower than those with trochanteric fractures (159.27 ± 5.20 *vs* 157.17 ± 4.11, 23.73 ± 1.56 *vs* 24.34 ± 1.27, respectively).

**Table 1:** Baseline characteristics among patients with cervical and trochanteric fractures (mean ± standard deviation)

Parameters	Cervical (n = 109)	Trochanteric (n = 87)	*P*‐value
Male	36	34	
Female	73	53	0.380
Age (years)	75.00 ± 9.47	78.72 ± 8.49	0.005*
Male	73.97 ± 10.65	77.44 ± 9.36	0.153
Female	75.51 ± 8.87	79.56 ± 7.86	0.009*
Menopausal age (years)	51.02 ± 2.57	50.36 ± 2.19	0.226
Height (cm)	163.23 ± 7.58	162.50 ± 8.08	0.513
Male	171.25 ± 4.77	170.80 ± 5.12	0.703
Female	159.27 ± 5.20	157.17 ± 4.11	0.016*
Weight (kg)	60.83 ± 12.53	59.45 ± 11.86	0.448
Male	66.74 ± 12.93	64.85 ± 12.03	0.531
Female	57.92 ± 11.31	56.06 ± 10.48	0.349
Body mass index (kg/m^2^)	22.66 ± 2.09	22.89 ± 2.21	0.470
Male	20.51 ± 1.16	20.62 ± 1.26	0.686
Female	23.73 ± 1.56	24.34 ± 1.27	0.021*

* Statistical significance was considered when *P* < 0.05.

### *Differences Between Cervical and Trochanteric Fractures in Terms of Bone Mineral Density and Hip Structural Analysis*

There was a significant difference between the two types of hip fracture in terms of hip BMD and HSA (Table 2). The hip BMD in the trochanteric fracture group was significantly lower in five locations than in the cervical fracture group. Then, male and female subgroups were assessed, and similar results were observed (Table 3 and Table 4). The hip BMD in both men and women with trochanteric fractures was lower than in those with cervical fractures. Furthermore, CSA, CortThick, and BR, as assessed through HSA, also differed between the two groups, whereby CSA and CortThick at the FN and IT sites of trochanteric fractures showed a significant decrease compared with those of cervical fractures. The BR results demonstrated a contrasting trend, whereby the BR was higher in trochanteric fractures compared with cervical fractures.

**Table 2:** Comparison between bone mineral density and hip structural variables among cervical and trochanteric fractures (mean ± standard deviation)

Parameters	Cervical (n = 109)	Trochanteric (n = 87)	*P*‐value
NeckBMD	0.591 ± 0.130	0.532 ± 0.115	0.001*
TrBMD	0.548 ± 0.103	0.494 ± 0.099	0.000*
InnerBMD	0.827 ± 0.177	0.765 ± 0.186	0.018*
TotalBMD	0.705 ± 0.138	0.653 ± 0.144	0.011*
WardBMD	0.417 ± 0.145	0.353 ± 0.125	0.001*
FNSubPeriwidth	3.81 ± 0.38	3.80 ± 0.46	0.888
FNEndoCortWidth	3.56 ± 0.39	3.57 ± 0.47	0.766
FNCSA	2.40 ± 0.59	2.17 ± 0.53	0.005*
FNCSMI	2.45 ± 0.98	2.34 ± 1.21	0.485
FNCortThick	0.14 ± 0.14	0.11 ± 0.04	0.084
FNBR	18.52 ± 5.05	20.56 ± 7.75	0.027*
ITSubPeriWidth	5.91 ± 0.66	5.80 ± 0.74	0.194
ITEndoCortWidth	5.34 ± 0.63	5.24 ± 0.82	0.339
ITCSA	3.90 ± 1.02	3.40 ± 0.99	0.001*
ITCSMI	11.83 ± 4.96	9.72 ± 4.61	0.003*
ITCortThick	0.29 ± 0.09	0.25 ± 0.08	0.006*
ITBR	12.82 ± 4.36	14.30 ± 4.63	0.023*
FSSubPeriWidth	3.25 ± 0.62	3.17 ± 0.51	0.587
FSEndoCortWidth	2.41 ± 0.80	2.33 ± 0.68	0.919
FSCSA	3.60 ± 0.86	3.39 ± 0.92	0.098
FSCSMI	3.60 ± 1.62	3.22 ± 1.32	0.087
FSCortThick	0.42 ± 0.14	0.41 ± 0.15	0.640
FSBR	4.92 ± 2.97	4.80 ± 2.43	0.763
Angle	129.30 ± 8.56	130.31 ± 6.73	0.867
HAL	103.17 ± 10.30	101.97 ± 9.50	0.402

Bone mineral density (BMD) was measured at five parts of the hip, including the femoral neck (NeckBMD), trochanter (TrBMD), inner (InnerBMD), Ward’s triangle (WardBMD), and total hip (TotalBMD). Hip structural analysis (HSA) was performed across the cross‐section of the femoral neck (FN), trochanteric region (IT), and femoral shaft (FS). Hip structural variables were assessed, including subperiosteal width (SubPeriWidth), estimated endosteal width (EndoCortWidth), cross‐sectional area (CSA), cross‐sectional moment of inertia (CSMI), estimated cortical thickness (CortThick), buckling ratio (BR), neck shaft angle (NSA), and hip axis length (HAL)

* Statistical significance was considered when *P* < 0.05.

**Table 3:** Comparison between bone mineral density and hip structural variables among cervical and trochanteric fractures in men (mean ± standard deviation)

Parameters	Cervical (n = 109)	Trochanteric (n = 87)	*P*‐value
NeckBMD	0.659 ± 0.134	0.596 ± 0.113	0.040*
TrBMD	0.605 ± 0.104	0.528 ± 0.104	0.003*
InnerBMD	0.924 ± 0.169	0.846 ± 0.189	0.070
TotalBMD	0.785 ± 0.143	0.713 ± 0.144	0.039*
WardBMD	0.496 ± 0.156	0.407 ± 0.118	0.007*
FNSubPeriwidth	3.92 ± 0.35	3.98 ± 0.35	0.526
FNEndoCortWidth	3.63 ± 0.35	3.74 ± 0.35	0.246
FNCSA	2.78 ± 0.68	2.60 ± 0.44	0.226
FNCSMI	3.16 ± 1.13	3.29 ± 1.33	0.667
FNCortThick	0.14 ± 0.03	0.12 ± 0.04	0.027*
FNBR	16.73 ± 4.15	18.54 ± 4.27	0.076
ITSubPeriWidth	6.30 ± 0.57	6.34 ± 0.71	0.791
ITEndoCortWidth	5.64 ± 0.57	5.80 ± 0.70	0.360
ITCSA	4.64 ± 0.97	4.04 ± 0.98	0.013*
ITCSMI	15.02 ± 5.30	13.24 ± 4.91	0.150
ITCortThick	0.33 ± 0.09	0.28 ± 0.09	0.027*
ITBR	11.65 ± 3.90	14.25 ± 4.82	0.015*
FSSubPeriWidth	3.44 ± 0.69	3.50 ± 0.54	0.378
FSEndoCortWidth	2.48 ± 0.95	2.58 ± 0.82	0.640
FSCSA	4.27 ± 0.82	4.07 ± 0.84	0.314
FSCSMI	4.65 ± 1.83	4.46 ± 1.19	0.897
FSCortThick	0.49 ± 0.15	0.46 ± 0.17	0.459
FSBR	4.45 ± 2.59	5.00 ± 2.79	0.412
Angle	130.31 ± 7.70	130.74 ± 7.44	0.813
HAL	110.17 ± 6.65	109.03 ± 7.50	0.503

Bone mineral density (BMD) was measured at five parts of the hip, including the femoral neck (NeckBMD), trochanter (TrBMD), inner (InnerBMD), Ward’s triangle (WardBMD) and total hip (TotalBMD). Hip structural analysis (HSA) was performed across the cross‐section of the femoral neck (FN), trochanteric region (IT) and femoral shaft (FS). Hip structural variables were assessed, including subperiosteal width (SubPeriWidth), estimated endosteal width (EndoCortWidth), cross‐sectional area (CSA), cross‐sectional moment of inertia (CSMI), estimated cortical thickness (CortThick), buckling ratio (BR), neck shaft angle (NSA) and hip axis length (HAL)

* Statistical significance was considered when *P* < 0.05

**Table 4:** Comparison between BMD and hip structural variables among cervical and trochanteric fractures in women (mean ± standard deviation)

Parameters	Cervical (n = 109)	Trochanteric (n = 87)	*P*‐value
NeckBMD	0.559 ± 0.114	0.491 ± 0.096	0.001*
TrBMD	0.520 ± 0.091	0.472 ± 0.089	0.004*
InnerBMD	0.778 ± 0.160	0.713 ± 0.166	0.027*
TotalBMD	0.666 ± 0.118	0.614 ± 0.132	0.023*
WardBMD	0.378 ± 0.121	0.317 ± 0.117	0.006*
FNSubPeriwidth	3.75 ± 0.38	3.67 ± 0.48	0.219
FNEndoCortWidth	3.52 ± 0.40	3.46 ± 0.51	0.408
FNCSA	2.21 ± 0.44	1.91 ± 0.40	0.000*
FNCSMI	2.10 ± 0.66	1.74 ± 0.57	0.002*
FNCortThick	0.14 ± 0.16	0.11 ± 0.03	0.002*
FNBR	19.40 ± 5.24	21.86 ± 9.13	0.150
ITSubPeriWidth	5.71 ± 0.62	5.42 ± 0.51	0.005*
ITEndoCortWidth	5.19 ± 0.61	4.89 ± 0.69	0.012*
ITCSA	3.50 ± 0.81	2.95 ± 0.73	0.010*
ITCSMI	10.26 ± 3.96	7.46 ± 2.54	0.000*
ITCortThick	0.27 ± 0.08	0.24 ± 0.07	0.028*
ITBR	13.40 ± 4.48	14.33 ± 4.55	0.254
FSSubPeriWidth	3.16 ± 0.57	2.96 ± 0.36	0.050
FSEndoCortWidth	2.37 ± 0.73	2.17 ± 0.53	0.319
FSCSA	3.27 ± 0.66	2.95 ± 0.68	0.009*
FSCSMI	3.08 ± 1.21	2.44 ± 0.59	0.001*
FSCortThick	0.39 ± 0.13	0.39 ± 0.13	0.756
FSBR	5.13 ± 4.66	4.66 ± 2.20	0.767
Angle	128.81 ± 8.97	130.04 ± 6.28	0.740
HAL	99.71 ± 10.05	97.43 ± 7.70	0.077

Bone mineral density (BMD) was measured at five parts of the hip, including the femoral neck (NeckBMD), trochanter (TrBMD), inner (InnerBMD), Ward’s triangle (WardBMD) and total hip (TotalBMD). Hip structural analysis (HSA) was performed across the cross‐section of the femoral neck (FN), the trochanteric region (IT) and the femoral shaft (FS). Hip structural variables were assessed, including subperiosteal width (SubPeriWidth), estimated endosteal width (EndoCortWidth), cross‐sectional area (CSA), cross‐sectional moment of inertia (CSMI), estimated cortical thickness (CortThick), buckling ratio (BR), neck shaft angle (NSA), and hip axis length (HAL)

* Statistical significance was considered when *P* < 0.05.

### *Risk Factors in Cervical and Trochanteric Fractures*

Through a backward stepwise logistic regression (Table 5), we found that age (/10 years), FNCSMI, and ITBR are significant risk factors for trochanteric fractures (OR 1.597, 95% CI 1.145–2.228, *P* = 0.006; OR 2.066, 95% CI 1.099–3.885, *P* = 0.024; OR 1.324, 95% CI 1.120–1.566, *P* = 0.001, respectively). In contrast, patients with higher bone mineral density of trochanter (TrBMD), FNCSA, and FSBR were at lower risk for trochanteric fractures (OR 0.005, 95% CI 0.000–0.094, *P* = 0.000; OR 0.179, 95% CI 0.048–0.662, *P* = 0.010; OR 0.668, 95% CI 0.463–0.965, *P* = 0.032, respectively). When we assessed male and female subgroups, we found that age (/10 year) is still a significant risk factor for trochanteric fractures in women (OR 1.599, 95% CI 1.046–2.771, *P* = 0.032). ITBR is a risk factor in men, and men with higher TrBMD and women with higher ITCSMI and FSBR are at lower risk for trochanteric fractures.

**Table 5:** Risk factors for trochanteric fractures compared with cervical fractures by logistic regression

	Parameter	Odds ratio	95% Confidence interval	*P*‐value
Total	Age (/10 year)	1.597	1.145–2.228	0.006
	TrBMD	0.005	0.000–0.094	0.000
	FNCSA	0.179	0.048–0.662	0.010
	FNCSMI	2.066	1.099–3.885	0.024
	ITBR	1.324	1.120–1.566	0.001
	FSBR	0.668	0.463–0.965	0.032
Male	TrBMD	0.001	0.000–0.126	0.006
	ITBR	1.337	1.093–1.636	0.005
Female	Age (/10 year)	1.599	1.046–2.771	0.032
	ITCSMI	0.640	0.454–0.902	0.011
	FSBR	0.611	0.408–0.916	0.017

Age (/10 year): mean every 10 years. BR, buckling ratio; CSA, cross‐sectional area; CSMI, cross‐sectional moment of inertia; FN, femoral neck; FS, femoral shaft; IT, trochanteric region; TrBMD, bone mineral density of trochanter.

## Discussion

With the aging process and the occurrence of osteoporosis, hip fractures have received more attention. Based on anatomical form and location, hip fractures can be classified into cervical and trochanteric fractures. In recent decades, several studies have indicated that women with trochanteric fractures are older, thinner, shorter, and have less bone mass at the proximal femur^12^, ^14^, ^21^, ^22^, ^23^. In the current study, we found that the average age of patients with trochanteric fractures was approximately 4 years older than that of patients with cervical fractures, and in female patients, those with trochanteric fractures were approximately 5 years older. This result is consistent with previous research^15^. We also found that female cervical fracture patients had a lower BMI and greater height compared with female trochanteric fracture patients. Mautalen et al^22^. reported that the average height of patients with trochanteric fractures was 4 or 5 cm less than that of patients with cervical fractures. In addition, the risk of fracture increased significantly with lower BMI, whereby a BMI of 20 kg/m^2^ was associated with a nearly twofold increase in risk ratio (RR = 1.95; 95% CI 1.71–2.22) compared with a BMI of 25 kg/m^2^ for hip fracture^24^. To sum up, we speculate that taller women with low BMI are prone to cervical fracture because of their unique anatomical structure (long HAL) and because they have less soft tissue protection (high buffer stress).

The current study has shown that there was a significant difference in BMD and HSA between cervical and trochanteric fractures. Cancellous bone is more abundant in the trochanter than in the femoral neck, and the trochanteric area consists of approximately 70%–90% trabecular bone. Consequently, BMD better predicts trochanteric than cervical fractures^25^. In the current study, the BMD in trochanteric fractures was markedly lower than that in cervical fractures at all five sites, by an approximate reduction of 10%. Similar results were found in male and female subgroups, whereby BMD decreased by 11.5% and 10.7%, respectively. Greenspan SL et al^26^. have reported that the BMD in the trochanteric area was 13% lower in women and 11% lower in men for patients with trochanteric fractures compared with those with femoral neck fractures. Therefore, the current findings have effectively corroborated those of previous investigations in Caucasian women and demonstrated that Asian women with lower BMD are also more likely to suffer trochanteric fractures.

However, because of structural differences in the hip, even patients with a relatively high BMD may suffer a femoral neck fracture. Hip geometry also significantly affects bone strength^27^, ^28^. A previous study showed that the risk of trochanteric fractures was strongly influenced by BMD, whereas the risk of cervical fracture was more influenced by mechanical factors^29^. In the current study, HSA was performed across the cross‐section at three different sites, including the FN, IT, and FS regions, and we found that there were some differences between the two types of hip fracture in the FN and IT regions. As shown in Table 5, the FNCSA, ITCSA, ITCSMI, and ITCortthick in the cervical fracture group are significantly higher than those in the trochanteric fracture group. As parameters of bone rigidity and geometry, CSA and CSMI represent bone mechanical strength and buckling strength, respectively. The larger the value of those parameters, the greater the mechanical strength of the bone. However, the BR of both the FN and IT regions was significantly lower in the cervical fracture group. BR, an index of cortical instability representing the ratio of the outer radius to the cortical thickness, was assessed as a measure of bone fragility^30^. The larger the BR value, the lower the mechanical strength and stability of the bones and the more vulnerable the skeleton is to breakage under external forces. Furthermore, the cortical bone mainly contributes to bone strength. The strength of the femoral neck will decline by less than 10% if all cancellous bone is removed^31^, and cortical thickness in the intertrochanteric region is very thin^32^. This confirms that cortical bone plays a major role in strengthening the proximal femur, particularly cortical thickness at the IT region. The current results indicate that trochanteric fracture patients with low bone strength and thin cortical thickness are more prone to fracture when they suffer from an external impact compared with cervical fracture patients. Furthermore, when men and women were assessed separately, similar results were also found, particularly in women. Compared with cervical fractures, there was a certain decrease in bone strength at three different sites (FN, IT, and FS) in trochanteric fractures. Moreover, there was also a significant decline in cortical thickness at both the FN and IT regions. Therefore, we speculate that the differences in bone structure in women that were more significant between the two groups may be caused by the influence of age and menopause. Pulkkinen et al^33^. found that patients with femoral neck fractures usually have more than a threefold greater NSA than patients with trochanteric fractures. However, we did not find a difference in HAL and NSA between the two types of hip fracture, and the same results have been reported by Maeda et al^12^, ^15^, ^32^, ^34^. We presume that the different results reported in these studies originate from differences in race, measurements, and small numbers of subjects. In addition, the HAL in Asian patients is short compared with that of other races^16^, making it difficult to find differences.

The OR of each variable was measured in the present study. Age, FNCSMI, and ITBR were risk factors in trochanteric fractures compared with cervical fractures. In contrast, TrBMD, FNCSA, and FSBR were protective factors. After adjustment for gender, we found that age and ITBR were still risk factors in trochanteric fractures compared with cervical fractures in women and men, respectively. TrBMD was a protective factor in men, as were ITCSMI and FSBR in women. We consider that the risk of trochanteric fractures was strongly influenced by age and BMD, whereas the risk of a cervical fracture was more influenced by mechanical factors.

This study has several limitations. The study design is cross‐sectional and employs a retrospective design, which could result in selection bias. However, by using strict inclusion and exclusion criteria, we can significantly reduce the selection deviation. Hip geometry was estimated from 2‐D DXA images, which could result in errors in HSA. Therefore, a standard position was used and the scanned image met strict criteria, which effectively reduced errors.

### *Conclusion*

The results of this study indicate that BMD and structural parameters were different in two types of hip fracture in elderly Chinese patients. Compared with cervical hip fracture patients, patients with trochanteric fractures were older, and demonstrated a lower BMD and less bone mechanical strength, especially in female patients. Age, FNCSMI, and ITBR were stronger risk factors for trochanteric hip fractures than for cervical fractures. Therefore, the fracture type should be considered in clinical fracture risk assessment and in studies related to fracture prevention.
